# Taste, A New Incentive to Switch to (*R*)-Praziquantel in Schistosomiasis Treatment

**DOI:** 10.1371/journal.pntd.0000357

**Published:** 2009-01-13

**Authors:** Thorsten Meyer, Harald Sekljic, Stefan Fuchs, Heiko Bothe, Dieter Schollmeyer, Christian Miculka

**Affiliations:** 1 Intervet Innovation GmbH, Schwabenheim, Germany; 2 Institute of Organic Chemistry, University of Mainz, Mainz, Germany; Institute of Cell Biology, Italy

## Abstract

**Background:**

Praziquantel (PZQ) is the drug compound of choice in the control and treatment of schistosomiasis. PZQ is administered as a racemate, i. e. 1∶1 mixture of enantiomers. The schistosomicidal activity arises from one PZQ-enantiomer, whereas the other enantiomer does not contribute to the activity. The WHO's Special Programme for Research and Training in Tropical Diseases (TDR) has assigned the low-cost preparation of pure schistosomicidal (−)-PZQ a key priority for future R&D on PZQ, but so far this transition has not happened. PZQ has two major administration drawbacks, the first being the high dose needed, and its well documented bitter and disgusting taste. Attempts of taste-masking by low-cost means have not been successful. We hypothesized that the non-schistosomicidal component in PZQ would be the main contributor to the unpleasant taste of the drug. If the hypothesis was confirmed, the two major administration drawbacks of PZQ, the high dose needed and its bitter taste, could be addressed in one go by removing the component contributing to the bitter taste.

**Methods and Findings:**

PZQ was separated into its schistosomicidal and the non-schistosomicidal component, the absolute stereochemical configuration of (−)-PZQ was determined to be (*R*)-PZQ by X-ray crystallography, and the extent of bitterness was determined for regular racemic PZQ and the schistosomicidal component in a taste study in humans. Finding: The schistosomicidal component alone is significantly less bitter than regular, racemic PZQ.

**Conclusion:**

Our hypothesis is confirmed. We propose to use only the pure schistosomicidal component of PZQ, offering the advantage of halving the dose and expectedly improving the compliance due to the removal of the bitter taste. Therefore, (*R*)-PZQ should be specifically suitable for the treatment of school-age children against schistosomiasis. With this finding, we would like to offer an additional incentive to the TDR's recommendation to switch to the pure schistosomicidal (*R*)-PZQ.

## Author Summary

Schistosomiasis, or Bilharzia, is a parasitic disease caused by flatworms, which affects about 200 million people worldwide. Praziquantel (PZQ) is the drug compound of choice in the control and treatment of this disease. Only half of the drug dose currently administered actually has activity against schistosomiasis, whereas the other half has no activity. Therefore, the WHO has assigned the low-cost preparation of the pure active component a key priority for future PZQ research and development. PZQ has two major administration drawbacks, the first being the high dose needed, the second its well documented bitter taste. Attempts of masking the unpleasant taste have not been successful. We hypothesized that the non-active component in PZQ would be the main contributor to the unpleasant taste of the drug. We determined the extent of bitterness for regular PZQ compared to the pure active component in a taste study in humans. We found that the pure active component alone is significantly less bitter than regular PZQ. This new finding should serve as an additional incentive for the PZQ research and development community to provide a low-cost, large-scale preparation route to the pure active component of PZQ.

## Introduction

Praziquantel [1] (PZQ) is the drug compound of choice in the control and treatment of schistosomiasis [2], in fact, it is the only commercially readily available drug. So far, no backup compound for PZQ of comparable efficacy and breadth of application is available. Clinically relevant resistance has not been observed, however differences in responses of PZQ-resistant and -susceptible *Schistosoma mansoni* to PZQ in vitro have been described [3]. PZQ is included in the WHO Model List of Essential Drugs [4] and is at the core of numerous schistosomiasis control programmes. The WHO's strategy for schistosomiasis control [5] aims at reducing morbidity through treatment with PZQ, with a focus on periodic treatment of school-age children and adults considered to be at risk. School-age children are seen as a high-risk group for schistosome infections because they are more susceptible to infection in cases where their increased nutritional needs are not adequately met, might be compromised by helminth infections in their cognitive development, and are continuously exposed to contaminated soil and water but probably less aware of the need for good personal hygiene [6].

While the safety and efficacy against all schistosoma species are outstanding, PZQ has two major administration drawbacks, the first being the high dose needed, 40 mg PZQ/kg bodyweight: Dosages in children are determined by measurement of children's heights using tablet poles, and range from one to five 600 mg-tablets for one treatment. Especially young children have been reported not to be able to swallow these 600 mg tablets [7]. The second drawback is PZQ's well documented bitter and disgusting taste, which can lead to gagging or vomiting if tablets are chewed contrary to recommendation [8]. In veterinary medicine, the oral delivery of PZQ to taste-sensitive companion animals like cats is known to be a challenge. Traditional methods of taste-masking, like the addition of aromas or sugar, are ineffective for PZQ. The bitterness of PZQ even led to PZQ's use as a bitter model drug compound in the effectiveness testing of sophisticated and expensive taste-masking techniques like micro-encapsulation [9] or drug active coating [10]. Apart from anecdotal evidence [2], we are not aware of reports of low compliance among children treated within schistosoma programmes due to the bitter taste. However, we have to assume that the unpleasant taste of PZQ does not lead to a treatment situation which school-age children would enjoy.

**Figure 1:**
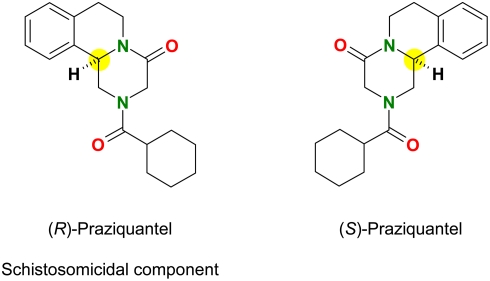
Molecular structures of the two mirror-image components of PZQ; asymmetric carbon atom highlighted in yellow.

PZQ is administered as a racemate, i. e. 1∶1, mixture of two compounds of identical constitution but non-superimposable mirror-image configuration, so called enantiomers. The straightforward and low-cost chemical synthesis has to be assumed as the reason for the use of the racemate, although it has been known for years that the schistosomicidal activity mainly relies in one PZQ-enantiomer, designated (−)-PZQ (alternatively termed levo-PZQ, l-PZQ, sometimes L-PZQ), whereas the other enantiomer, designated (+)-PZQ (alternatively termed dextro-PZQ, d-PZQ), does not contribute to the activity [11]–[13] (Figure 1). From this perspective, only half of the drug compound administered is in fact the drug active, whereas the other half must be considered molecular ballast, which has to be metabolized and excreted while not contributing to the schistosomicidal activity. To the best of our knowledge, no clinical studies in humans exist if and how non-schistosomicidal (+)-PZQ alone contributes to the side effects known of racemic PZQ, but this may be assumed: Upon incubation of PZQ and both enantiomers with isolated rat hepatocytes, additional metabolites were detected resulting from the non-contributing (+)-PZQ [14]. Various methods of producing the pure schistosomicidal component (−)-PZQ exist, which are considerably more expensive than the production of racemic PZQ itself. So far, the potential alone to administer half the current dose by replacing racemic PZQ by (−)-PZQ did not lead to a production process for (−)-PZQ comparable in costs for racemic PZQ.

In the context of the WHO's Global Plan to combat NTDs [15], the Special Programme for Research and Training in Tropical Diseases (TDR) set up an incentive for further R&D work by emphasizing the low-cost preparation of pure schistosomicidal (−)-PZQ (see also the schistosomiasis research collaborative community within *The Synaptic Leap* [16]) as a key priority for future R&D on PZQ [17]. Three pharmacological goals for the development were stated: (1) same dose of (−)-PZQ as currently in regular, racemic PZQ, with smaller tablet size and less frequent/severe adverse events, (2) higher dose of (−)-PZQ with similar tablet size and possibly similar adverse event profile as current treatment which could reduce the probability of or delay the development of resistance, or (3) a combination of these two objectives. As we already mentioned, a smaller tablet size would be more suitable for the treatment of children. Taking into account that the WHO's strategy specifically aims at school-age children, we were intrigued by the question whether the taste disadvantage of PZQ could be turned into an additional incentive to introduce (−)-PZQ against schistosomiasis as the drug active of choice. Background to our consideration was the well-documented fact that in most cases taste experiences depend on the stereochemical configuration of the agent [18], i. e. the taste buds react enantioselectively–like all natural receptors which are composed of chiral constituents like L-amino acids. We hypothesized that (−)-PZQ and (+)-PZQ would contribute to the bitter taste to a different extent, and that the non-schistosomicidal (+)-PZQ would be the main or sole contributor to the disgusting taste. Surprisingly, no public knowledge exists on the tastes of the two enantiomers. We prepared schistosomicidal (−)-PZQ, assigned the stereochemical configuration by X-ray crystallography, and determined the extent of bitterness for regular racemic PZQ versus the schistosomicidal component (−)-PZQ in a taste study in humans. We chose this comparison over the comparison of (−)-PZQ to non-schistosomicidal (+)-PZQ because the latter alone does not have any role in a treatment situation. Also the pharmacological studies by others had compared racemic PZQ to (−)-PZQ, and not (+)-PZQ to (−)-PZQ [19].

## Methods

### Preparation and stereochemical assignment of (−)-PZQ

**Figure 2:**
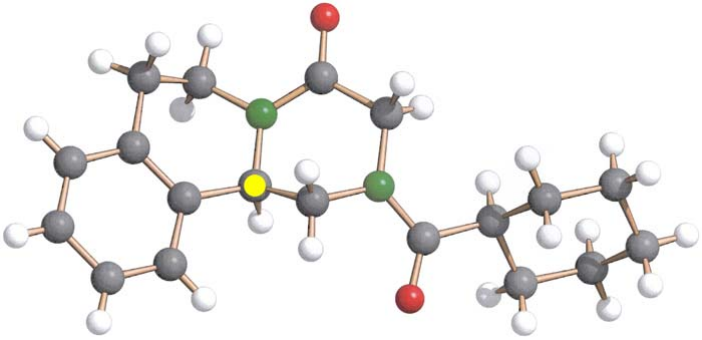
X-ray crystallographic structure of (*R)*-PZQ; asymmetric carbon atom highlighted in yellow.

Although effective synthetic methods for the enantioselective preparation of PZQ have been reported [20], we opted for the direct enantioseparation of the racemate yielding gram quantities of both optical forms. The preparative scale chromatography was performed on microcrystalline cellulose triacetate using methanol as the mobile phase, conditions under which the enantiomer having the negative optical rotation emerged first from the column [21]. After crystallisation from methanol/water, (−)-PZQ was obtained in enantiomeric excess >99%, as determined by HPLC (column used Chiralcel OD-H). No residual other enantiomer (+)-PZQ was detected in this sample. X-ray structural analysis, using Cu-Kα radiation, of a monoclinic crystal in hemi-hydrate form obtained from said fraction by crystallization from methanol/water unequivocally proved the *R*-configuration of the molecule by measuring Friedel pairs and the Flack parameter (x = −0.1(3)) (Figure 2). Further details of the crystal structure analysis are available on request from the CCDC (www.ccdc.cam.ac.uk) quoting the names of the authors and journal citation.

### Determination of the bitterness value

The bitterness values of racemic PZQ and its schistosomicidal component (*R*)-PZQ were determined according to the European Pharmacopoeia [22] by comparison with quinine hydrochloride, the bitterness value of which is set at 2×10^5^. The bitterness value is defined by the European Pharmacopoeia as the reciprocal of the concentration of a solution in a dilution series of a compound, a liquid or an extract that still has a bitter taste. Concentrations of solutions used in the tests ranged from 1.69×10^−8^ to 1.0×10^−4^ g/mL. A test panel consisting of sixteen members was assembled. Although children comprise the treatment target group no children were included in the test panel. All panel members were adults completely untrained in performing sensory tests. To correct for individual differences in tasting bitterness amongst the panel members a correction factor was determined for each panel member by preparing dilutions of quinine hydrochloride. The mouth was rinsed with water before tasting. The dilution with the lowest concentration having a bitter taste was determined by taking 10 mL of the weakest solution into the mouth and passing it from side to side over the back of the tongue for 30 seconds. If the solution was not found to be bitter, the panellist had to spit out and wait for one minute before the mouth was rinsed again with water. After 10 minutes, the next dilution in order of increasing concentration was tasted. The correction factor *k* for each panel member was calculated according to the European Pharmacopoeia by *k* = *n*/5, where *n* is the number of millilitres of the stock solution in the dilution of the lowest concentration that is judged to be bitter. One panel member detected bitterness already in pure water, and was therefore excluded from the test panel. Dilutions of the test compounds racemic PZQ and (*R*)-PZQ were prepared and tasted by the remaining fifteen members of the test panel in the same manner as described for quinine hydrochloride. The bitterness value as experienced by each member was calculated according to the European Pharmacopoeia taking the individual-related correction factor into account by *Y*×*k*/*X*×0.1, where *Y* is the dilution factor of the dilution, and *X* is the number of millilitres of the respective dilution which, when diluted to 10 mL with water, still has a bitter taste. The bitterness value of the test compounds resulted from calculating the average of the individual values.

Requested statement: Informed written consent was obtained from all panelists to participate in this taste study. As a taste study, and not a medical study in the sense of the WMA Declaration of Helsinki, it did not require approval of an independent review board (highly diluted preparations were tasted and spat out–they were not ingested). Nevertheless, it was conducted according to the principles of the WMA Declaration of Helsinki where applicable.

## Results

**Figure 3:**
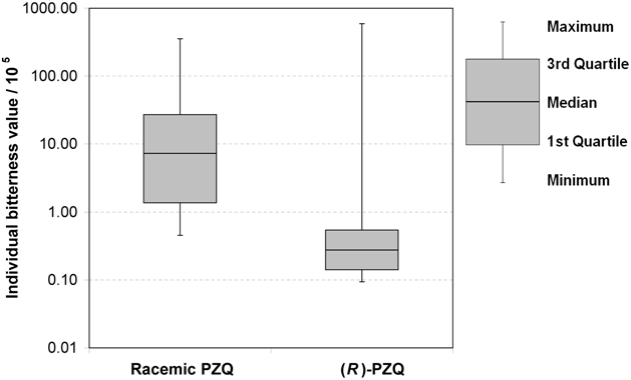
Box-and-whisker diagram of the individual bitterness values for racemic PZQ and (*R*)-PZQ.

The results of the determination of bitterness values are shown in Table 1. Remarkable is the variation of the individuals' results as indicated by the relative standard deviation and the dispersion of the results in the box-and-whisker diagram (Figure 3). In contrast to the average, the medians of the results, as depicted in the box-and-whisker diagram, are different from each other. The observed variation was probably provoked by the test panel consisting of untrained members only [23]. Thirteen out of fifteen panel members found (*R*)-PZQ to taste less bitter than racemic PZQ. Although no statistical test is required or proposed by the European Pharmacopoeia, a statistical test (using SAS software, release 9.1.3, SAS Institute Inc., Cary, NC, USA) was conducted to investigate the observed difference between the compounds. Considering the small sample size and the nature of the data which does not justify the assumption of a normal distribution, a nonparametric, distribution-free method was chosen. On the 5% level of significance, Wilcoxon's Signed Rank Test (two-sided) resulted in a significant difference between the taste of racemic PZQ and (*R*)-PZQ (p = 0.0107). This result was confirmed by the Sign Test (two-sided, p = 0.0018).

**Table 1:** Bitterness values for racemic PZQ and schistosomicidal (*R*)-PZQ

Member of the test panel	Racemic PZQ	(*R*)-PZQ
	Individual bitterness values/10^5^	
A	1.03	0.14
B	0.46	0.11
C	7.85	0.68
D	7.85	0.09
E	1.92	1.23
F	1.71	0.27
G	355	592
H	177	178
I	250	0.37
J	0.62	0.25
K	14.5	0.29
L	39.4	0.10
M	5.52	0.41
N	7.23	0.14
O	0.55	0.28
Bitterness value (average)	58.1	51.6
Standard deviation	110	156
Relative standard deviation	191%	303%

In addition to the quantitative determination of the bitterness values, qualitative taste sensations were noted by the members of the test panel for each compound. For racemic PZQ, all panel members commonly observed the sensation of an unpleasant chemical or metallic taste or a taste circumscribed best by old rubber. On the other hand, for (*R*)-PZQ the panellists commonly described the sensation of a moderate chemical taste, comparable to that of a polyethylene or a rubber pipe. Although the tastes were not recognized alike across the test panel, for the majority of the test panel we can state that (*R*)-PZQ had a less unpleasant taste compared to racemic PZQ.

## Discussion

The schistosomicidal component of regular PZQ, (*R*)-PZQ has a less unpleasant taste compared to racemic PZQ, which was found to be comparably bitter or unpleasant. It can be assumed that the disgusting taste of racemic PZQ stems from the non-schistosomicidal component, (*S*)-PZQ. Removing the latter from currently used racemic PZQ therefore not only offers the chance to halve the dose, with the potential to decrease the number or size of the tablets, but also addresses the second disadvantage of regular, racemic PZQ-its unpleasant taste. With this finding and its publication we would like to offer an additional incentive to focus work of the PZQ R&D community on further decreasing the cost of production of (*R*)-PZQ with the goal to switch to pure (*R*)-PZQ as a replacement for racemic PZQ for the treatment of school-age children against schistosomiasis.
